# Prognostic Value of Serum Interleukin-6, NF-*κ*B plus MCP-1 Assay in Patients with Diabetic Nephropathy

**DOI:** 10.1155/2022/4428484

**Published:** 2022-06-17

**Authors:** Zhongwu An, Jibao Qin, Weibo Bo, Haiying Li, Ling Jiang, Xin Li, Jie Jiang

**Affiliations:** ^1^The Clinical Laboratory of Lianyungang Oriental Hospital, The Affiliated Lianyungang Oriental Hospital of Xuzhou Medical University, Lianyungang, China; ^2^The Department of Pharmacy of Lianyungang Oriental Hospital, The Affiliated Lianyungang Oriental Hospital of Xuzhou Medical University, China

## Abstract

**Objective:**

To assess the prognostic value of serum interleukin-6 (IL-6), nuclear factor-*κ*B (NF-*κ*B), and monocyte chemoattractant protein 1(MCP-1) assay in patients with diabetic nephropathy.

**Methods:**

From May 2019 to March 2020, 104 patients with diabetic nephropathy treated in our institution assessed for eligibility were recruited and assigned at a ratio of 1 : 1 to either the observation group ([urinary albumin excretion rate (UAER)] of 30 mg-300 mg/24 h) or the research group ([UAER] >300 mg/24 h). IL-6, MCP-1, renal function indices, and NF-*κ*B levels were determined, and their correlation with DN was analyzed. Logistic regression was used to analyze the influencing factors of end-stage renal disease in patients with diabetic nephropathy. The receiver operating characteristic (ROC) curve was drawn, and the area under the curve (AUC) was calculated to analyze the predictive value of combined detection of IL-6, MCP-1, and NF-*κ*B in the prognosis of patients with diabetic nephropathy.

**Results:**

The eligible patients with UAER of 30 mg-300 mg/24 h were associated with significantly higher levels of IL-6, MCP-1, NF-*κ*B, blood urea nitrogen (BUN), and serum creatinine (Scr) versus those with UAER >300 mg/24 h (*P* < 0.05). During the follow-up, a total of 38 patients progressed to end-stage renal diseases. Eligible patients with end-stage renal diseases showed significantly higher serum IL-6, MCP-1, and NF-*κ*B levels versus those without end-stage renal diseases (*P* < 0.05). Serum IL-6, MCP-1, and NF-*κ*B are independent risk factors for the occurrence of end-stage renal disease in patients with diabetic nephropathy. The AUCs of IL-6, MCP-1, and NF-*κ*B for predicting the prognosis of patients with diabetic nephropathy were 0.562, 0.634, and 0.647, respectively, and the AUC of the three combined detection for predicting the prognosis of patients with diabetic nephropathy was 0.889.

**Conclusion:**

Serum IL-6, NF-*κ*B, and MCP-1 levels are closely related to renal injury and poor prognosis in patients with diabetic nephropathy, and the combined assay is valuable for assessing patients' condition and prognosis.

## 1. Introduction

Diabetic nephropathy, a kidney disease associated with diabetes mellitus is one of the microvascular complications in diabetic patients and has become the second cause of terminal-stage renal disease worldwide, second only to glomerulonephritis, with a predisposition to combined macrovascular events. Diabetic nephropathy is associated with genetic background and risk factors and may lead to proteinuria, edema, hypertension, and even kidney failure, posing a great threat to the life safety of patients. Epidemiological studies have confirmed the prevalence of diabetic nephropathy up to about 273-482/10,000 people. The clinical development of diabetic nephropathy contributes to an increased incidence of mortality and end-stage renal failure in patients. Interleukin 6 (IL-6) is a cytokine produced by immune cells that facilitates the regulation and promotion of the immune response and stimulates the production of acutely relevant reactants, which shows a trend of increase in the blood in response to inflammation or tissue damage [1–4]. Nuclear factor-*κ*B (NF-*κ*B) is a key class of nuclear transcription factors that are usually present in the cytoplasm of almost all cell types in homo- or heterodimeric inactive form. It is involved in the activation of immune cells, development of T and B lymphocytes, stress response, cellular regulation, and many other cellular activities. Multiple factors activate and translocate NF-*κ*B from the cytoplasm to the nucleus, where it binds to the KB site of NF-*κ*B-responsive genes and regulates the transcription of NF-*κ*B-responsive genes [5–7]. Monocyte chemotactic protein 1 (MCP-1) belongs to a small cytokine of the CC chemokine family, which has been identified as an important proinflammatory cytokine secreted by monocytes, macrophages, fibroblasts, vascular endothelial cells, B cells, and smooth muscle cells during an inflammatory response. Moreover, MCP-1 has specific chemotactic activation on monocytes/macrophages [8–10]. Accordingly, the present study investigated the prognostic value of the combined assay of IL-6, NF-*κ*B, and MCP-1 in diabetic nephropathy to provide a reference for future clinical practice.

## 2. Materials and Methods

### 2.1. Baseline Data

From May 2019 to March 2020, 104 patients with diabetic nephropathy treated in our institution assessed for eligibility were recruited and assigned at a ratio of 1 : 1 to either the observation group ([Urinary albumin excretion rate] UAER of 30 mg-300 mg/24 h) or the research group (UAER >300 mg/24 h). The clinical baseline features of the observation group (aged 50-77 years, mean age of [62.31 ± 12.10] years, 29 males and 23 females, a mean body mass index (BMI) of [22.68 ± 3.12] kg/m^2^, and mean diabetes duration of (5.21 ± 1.38) years) were comparable with those of the research group (aged 51-77 years, mean age of [62.41 ± 12.02] years, 28 males and 24 females, a mean BMI of [22.49 ± 3.07] kg/m^2^, and a mean diabetes duration of (5.33 ± 1.45) years) (*P* > 0.05). The observation group had a significantly lower FPG (4.37 ± 1.21 vs. 5.68 ± 1.32) mmol/L and a significantly higher glycated hemoglobin (5.14 ± 1.13 vs. 4.67 ± 1.15) % than the research group. (Table 1). Patients provided written informed consent, and this study was approved by the medical ethics committee (Ethics No. 20190456).

### 2.2. Inclusion and Exclusion Criteria

Inclusion criteria were as follows: ① Diabetic patients were diagnosed as per the diagnostic criteria established by the World Health Organization Professional Committee on Diabetes in 1999: (a) fasting blood glucose ≥7.0 mmol/L; (b) random blood glucose ≥11.1 mmol/L; (c) OGTr 2 h blood glucose value ≥11.1 mmol/L; any one of the above three points can be diagnosed; ②Patients were diagnosed with nephropathy based on the clinical renal function assessment; ③ aged 19-79 years; and ④ Patients received no relevant treatment before enrollment.

Exclusion criteria were as follows: ① Patients with renal tumor; ② with severe psychiatric disease; ③ with hypertensive nephropathy, primary renal disease; ④ with cerebrovascular disease; ⑤ with hematologic disease; and ⑥ diabetic patients with renal insufficiency and without proteinuria.

### 2.3. Assay Method

Before treatment, 5 mL of fasting peripheral venous blood was collected from patients and stored in a 4°C refrigerator. IL-6, MCP-1, and NF-*κ*B monoclonal antibodies were added, respectively, followed by 5 min rinsing with PBS solution. 5 mL of the sheep anti-human primary antibody was then added at a ratio of 1 : 500, left overnight at 4°C, and washed 3 times with PBS buffer for 5 min each time followed by the addition of 2 mL of mouse-derived secondary antibody (1 : 1000), placed at room temperature for 2 h, and washed with PBS solution for 5 min. Color development was carried out by adding horseradish peroxidase, and a termination solution was added for the termination of the reaction. The optical density (OD) value was measured at 450 nm. IL-6, MCP-1, and NF-*κ*B kits were purchased from Wuhan Fien Biotechnology Co., Ltd (Item No. EH0201), Wuhan Fien Biotechnology Co., Ltd (Item No. EH0222), Shanghai Hengfei Biotechnology Co. (Item No. CSB-E12107h-1). The assay instrument was a SpectraMax iD5 enzyme marker from Molecular Devices, USA.

### 2.4. Assay of Blood Urea Nitrogen (BUN) and Blood Creatinine (Scr)

After randomization, 5 mL of peripheral venous blood was collected from patients in fasting state before treatment and stored in a refrigerator at 4°C. The supernatant was separated by centrifugation at 1000 r/min for 5 min with a radius of 10 cm. The BUN and Scr levels were determined using a fully automated biochemical method, the kits were purchased from Nanjing Biyuntian Bioassay Company, and the microcentrifuge HITETIC was purchased from Shanghai Precision Instruments Co.

### 2.5. Statistical Analysis

SPSS22.0 was used for data analyses, and GraphPad Prism 8 was used for image rendering. The measurement data are expressed as (−*x* ± *s*) and processed by the independent samples *t*-test. The count data are expressed as the number of cases (rate) and analyzed using the chi-square test. Logistic regression was used to analyze the influencing factors of end-stage renal disease in patients with diabetic nephropathy. The receiver operating characteristic (ROC) curve was drawn, and the area under the curve (AUC) was calculated to analyze the predictive value of combined detection of IL-6, MCP-1, and NF-*κ*B in the prognosis of patients with diabetic nephropathy. Differences were considered statistically significant at *P* < 0.05.

## 3. Results

### 3.1. IL-6, MCP-1, and NF-*κ*B

The levels of IL-6, MCP-1, and NF-*κ*B in the observation group were (6.23 ± 2.77) ng/L, (20.17 ± 5.32) *μ*g/L, and (24.78 ± 6.87) *μ*g/L, respectively, and the levels of IL-6, MCP-1, and NF-*κ*B in the research group were (10.88 ± 4.27) ng/L, (28.12 ± 6.02) *μ*g/L, and (52.29 ± 9.24) *μ*g/L, respectively. The levels of IL-6, MCP-1, and NF-*κ*B in the research group were higher than those in the observation group (*P* < 0.05). (Table1).

### 3.2. Renal Function Indices

**Figure 1:**
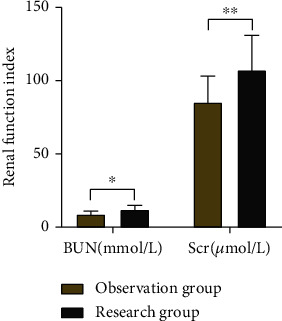
Comparison of renal function indices(−*x* ± *s*).

The BUN level was (8.82 ± 2.11) and Scr level was (85.15 ± 17.94) in the observation group, and the BUN level was (12.06 ± 2.90) and Scr level was (107.13 ± 23.82) in the research group. The research group had statistically higher BUN and Scr levels than the observation group (*P* < 0.05). (Figure 1).

### 3.3. Serum IL-6, NF-*κ*B, and MCP-1 Levels and Prognosis in Patients with Diabetic Nephropathy

During the follow-up, a total of 38 patients progressed to end-stage renal diseases. Eligible patients with end-stage renal diseases showed significantly higher serum IL-6, MCP-1, and NF-*κ*B levels versus those without end-stage renal diseases (*P* < 0.05).

### 3.4. Logistic Regression Analysis of Factors Affecting the Occurrence of End-Stage Renal Disease in Patients with Diabetic Nephropathy

Gender, age, BMI, course of disease, FPG, glycosylated hemoglobin, BUN, Scr, IL-6, MCP-1, and NF-*κ*B were used as single factors, and the occurrence of end-stage renal disease was the end point. Multivariate analysis showed that serum IL-6, MCP-1, and NF-*κ*B were independent risk factors for the development of end-stage renal disease in patients with diabetic nephropathy (Table 2).

### 3.5. Predictive Value of Combined Detection of IL-6, MCP-1, and NF-*κ*B in the Prognosis of Patients with Diabetic Nephropathy

The AUCs of IL-6, MCP-1, and NF-*κ*B for predicting the prognosis of patients with diabetic nephropathy were 0.562, 0.634, and 0.647, respectively, and the AUC of the three combined detection for predicting the prognosis of patients with diabetic nephropathy was 0.889 (Table 3).

## 4. Discussion

Diabetic nephropathy is one of the microvascular complications in diabetic patients and has become the second cause of end-stage renal disease worldwide, second only to glomerulonephritis, and is prone to macrovascular events. The proportion of diabetic patients with renal failure is increasing year by year, and the risk of end-stage renal failure increases with age. Diabetic nephropathy assessments provide an effective reference for clinical immunotherapy and hormone therapy, which promotes early clinical intervention and thus lays the foundation for the improvement of renal function in the long run. Urine protein or 24-hour urine protein quantification is the traditional index for DN disease assessment, which can effectively indicate the degree of renal impairment and glomerular filtration membrane damage. However, urine protein quantification is affected by dietary habits and metabolic consumption of patients, which is, therefore, considered deficient for early prediction. Serum markers such as IL-6 and MCP-1 are significantly altered in the early stages of kidney injury, and the detection of relevant molecular markers in peripheral blood provides a novel auxiliary reference for the clinical management of diabetic nephropathy. To date, some studies have explored the expression of MCP-1 in diabetic nephropathy patients and concluded that MCP-1 can influence the level of end-stage renal function in diabetic nephropathy patients; however, the specific exploration of IL-6 and MCP-1 in diabetic nephropathy patients with varying UAER levels is marginally explored [11–13].

IL-6 and MCP-1 affect the activation of inflammatory signaling pathways and promote the disruption of the charge barrier and physiological barrier of the renal filtration membrane, leading to leakage of urinary protein. The rise of MCP-1 in monocytes or macrophages can exacerbate the release of oxidoreductase, which leads to apoptosis of the filtration membrane foot cells and an increase of the filtration membrane pore size. Dong et al. concluded that in patients with diabetic nephropathy, serum IL-6 expression increases with the elevation of diabetic complications, especially in patients with lower limb microangiopathy or renal microangiopathy. The expression of IL-6 and MCP-1 increased more significantly in patients with diabetic nephropathy with more severe urinary protein and overall disease exacerbation. The leakage of urinary protein exacerbates the deterioration of the nutritional status of the body and induces damage to the renal tubules or collecting ducts, which leads to increased elevation of IL-6 and MCP-1. Basic clinical trials have shown that activation of the NF-*κ*B inflammatory signaling pathway is an important pathological mechanism in the development of diabetic nephropathy, and it maintains the stability of the renal internal environment and mediates pathogen-specific responses. At rest, NF-*κ*B exists as a p50/p65 dimer in the cytoplasm, and homo- and/or heterodimers formed by the two subunits bind to its inhibitory protein I*κ*B to form an inactive trimer. NF-*κ*B originating from inflammatory response activation stimulates the induction of IL-6 and MCP-1 secretion, which in turn activates I*κ*B kinase complexes (IKKs). IKKs phosphorylate the serine of the I*κ*B subunit regulatory site of the trimeric p50/p65/I*κ*B, resulting in ubiquitination modification of the I*κ*B subunit, the degradation by proteases, and subsequent release of p50/p65 and nuclear translocation to the *κ*B site on the corresponding target gene to regulate gene transcription [14–17]. IL-6 and MCP-1 can activate a large number of inflammatory cells, and the resulting inflammatory cascade amplification is associated with distant organ damages, which positively regulates the activation of NF-*κ*B and aggravates the damage to the intracellular and basement membranes, thereby eliciting proteinuria. IL-6 is mostly activated and released by the overexpression of NF-*κ*B, leading to the accumulation of neutrophils, basophils, and T cells in the kidney, which induces glomerular production of interstitial proteins to promote glomerular fibrosis, thereby contributing to the pathological damage of the kidney. In the present study, the significantly higher IL-6, MCP-1, and NF-*κ*B levels in the research group than in the observation group suggest the involvement of IL-6, MCP-1, and NF-*κ*B in the progression of diabetic nephropathy, which was consistent with the results of the previous research [18–21]. The levels of creatinine (Scr) and urea nitrogen (BUN) in the research group were significantly higher than those in the observation group; the more severe the patients' diabetic nephropathy, the higher their serum levels of Scr and BUN, which were mainly related to the patients' renal impairment due to long-term dysglycemia. Moreover, the research results of the present study demonstrated that during the follow-up, a total of 38 patients progressed to end-stage renal disease. Eligible patients with end-stage renal diseases showed significantly higher serum IL-6, MCP-1, and NF-*κ*B levels versus those without end-stage renal diseases, and multivariate analysis showed that serum IL-6, MCP-1, and NF-*κ*B were independent risk factors for the development of end-stage renal disease in patients with diabetic nephropathy. The AUCs of IL-6, MCP-1, and NF-*κ*B for predicting the prognosis of patients with diabetic nephropathy were 0.562, 0.634, and 0.647, respectively, and the AUC of the three combined detection for predicting the prognosis of patients with diabetic nephropathy was 0.889. This further confirmed a close correlation between serum IL-6, MCP-1, and NF-*κ*B levels and the prognosis of patients with diabetic nephropathy, which is of clinical value for the prognostic assessment of the disease.

To sum up, serum IL-6, NF-*κ*B, and MCP-1 levels are closely related to renal injury and poor prognosis in patients with diabetic nephropathy, and the combined assay is valuable for assessing patients' condition and prognosis.

## Figures and Tables

**Table 1:** Comparison of baseline features [*n* (%)].

	Observation group (*n* = 52)	Research group (*n* = 52)	*t* or *x*2	*P*
Mean age	62.31 ± 12.10	62.41 ± 12.02	-0.042	0.967
Gender			0.039	0.844
Male	29	28		
Female	23	24		
BMI (kg/m^2^)	22.68 ± 3.12	22.49 ± 3.07	0.313	0.755
Diabetes duration (yr.)	5.21 ± 1.38	5.33 ± 1.45	0.432	0.666
FPG (mmol/L)	4.37 ± 1.21	5.68 ± 1.32	5.275	≤0.001
Glycated hemoglobin (%)	5.14 ± 1.13	4.67 ± 1.15	2.102	0.038
IL-6 (ng/L)	6.23 ± 2.77	10.88 ± 4.27	6.588	≤0.001
MCP-1 (*μ*g/L)	20.17 ± 5.32	28.12 ± 6.02	7.136	≤0.001
NF-*κ*B (*μ*g/L)	24.78 ± 6.87	52.29 ± 9.24	17.229	≤0.001

**Table 2:** Logistic regression analysis of factors affecting the occurrence of end-stage renal disease in patients with diabetic nephropathy.

	*β*	SE	HR	95% CI	*P*
IL-6	1.053	0.231	1.934	1.328-1.997	0.011
MCP-1	1.257	0.345	1.836	1.382-1.935	0.024
NF-*κ*B	1.536	0.483	2.346	1.586-2.697	0.017

**Table 3:** Predictive value of combined detection of IL-6, MCP-1 and NF-*κ*B on the prognosis of patients with diabetic nephropathy.

	Sensitivity/%	Specificity/%	SE	*P*	AUC	95% CI
IL-6	63.82	76.38	0.034	≤0.001	0.562	0.458-0.679
MCP-1	64.28	77.75	0.042	≤0.001	0.634	0.573-0.712
NF-*κ*B	65.37	78.11	0.017	≤0.001	0.647	0.591-0.754
IL-6 + MCP-1 + NF-*κ*B	89.67	84.58	0.006	≤0.001	0.889	0.738-0.998

## Data Availability

The datasets used during the present study are available from the corresponding author upon reasonable request.
